# Understanding Opioid Use from Retrospective Accounts of Young Adults in Recovery: Motives, Experiences, and Implications for Prevention

**DOI:** 10.1007/s10935-025-00861-y

**Published:** 2025-06-27

**Authors:** Parissa J. Ballard, Elena M. Vidrascu, Taylor J. Arnold, Guadalupe C. Hernandez, Emily J. Ozer, Rebekah Lassiter, Himani Nayyar, Stephanie S. Daniel, Mark Wolfson

**Affiliations:** 1 Family & Community Medicine, Wake Forest University School of Medicine, 1920 West 1st St., Winston-Salem, NC 27104 USA; 2 Psychology & Neuroscience, University of North Carolina at Chapel Hill, 103 Howell Hall, Chapel-Hill, NC 27713 USA; 3 University of North Carolina, Gillings School of Global Public Health, Chapel-Hill, USA; 4 Community Health Sciences, University of California Berkeley, School of Public Health, Berkeley, USA; 5 Wake Forest University, Winston-Salem, NC USA; 6 Department of Social Medicine, Population, and Public Health, University of California, Riverside, USA

**Keywords:** Opioid use, Youth and young adults, Motives

## Abstract

Opioid use disorder is a public health problem with disastrous effects for young people and their families. Opioid use shares risk factors with other substances; there may be additional unique motives and experiences associated with opioid use with potential implications for improving prevention. The present study used surveys and interviews to elicit retrospective accounts of 30 young adults (19 female) in recovery from opioid use disorder to examine (1) self-reported motives for opioid use, and (2) experiences and factors that participants associate with opioid use as compared to other substances. Motives reported for past opioid use on surveys were *enhancement* (i.e., fun or excitement) and *coping* (i.e., to forget worries or cheer up when in a bad mood), followed by *conformity* and *social* motives. Through analysis of interview data, we found that: *experiences* of opioid use differed from experiences associated with other substances, with effects described in terms of escape and numbing (theme 1); and the ability (early in use) to remain/appear functional (theme 2). Participants reported perceptions of lower risk of harm compared to other substances (theme 3). For some, access was the main factor reported to contribute to opioid use (theme 4). Evidence-based prevention approaches focused on environmental strategies (such as prescription regulation policies) and individual strategies (such as coping skills and prevention education) are relevant for opioid use prevention. Additional supplemental approaches are needed to focus on misperceptions among young people that prescription medications confer lower risk than other substances.

Non-medical use of prescription opioids and illicit opioids such as heroin (hereafter referred to as “opioid use”) continues to be an urgent public health problem for adolescents and young adults (SAMHSA, 2021). The “opioid epidemic” has evolved through several phases; overdose deaths primarily resulted from use of prescription opioids in the 1990s, adulterated heroin supplies (e.g., fentanyl) in the 2010s, and synthetic opioids more recently (Baldwin et al., 2021; Cicero et al., 2017, FCC, n.d.a, FCC, n.d.b). Although recent prescription monitoring programs have helped to regulate prescribing of opioid analgesics by limiting the number of prescriptions (Cicero et al., 2017), additional opioid options have emerged in the illegal drug supply, therefore providing a more accessible and less expensive alternatives. An unintended consequence of the reduced misuse of prescription opioids is the increased availability and use of illegal opioids, many of which have increased potency carrying higher risk of subsequent overdose and deaths (Cicero et al., 2020). Opioids thus remain accessible to young people, highly addictive, and increasingly harmful as supply contamination issues have increased the risk of overdose. Adulterants such as methamphetamine, lidocaine, and xylazine, and impurities added during the manufacturing process of illicit opioids, have posed additional health risks that result from either direct toxicity or unknown synergistic reactions with opioids (Singh et al., 2020). Furthermore, despite nearly 10% of deaths among youth being attributed to opioid overdose, the treatment gap disproportionally affects youth in comparison to adults (Robinson & Wilson, 2020). The opioid crisis is a societal problem and systemic solutions are needed. However, despite heavy financial, academic, and public investment in curbing opioid use and addressing its negative effects on the lives of young people and families in the United States, the crisis remains.

While recent evidence has converged suggesting that there are more shared than unique risk factors for opioid use compared to other substances (Circero et al., 2020; Pandika et al., 2022), understanding potentially unique motives and experiences associated with opioid use can inform supplemental approaches or tailoring of established prevention strategies such as school-based programs like Safety First (Liu et al., 2025) and community-wide models such as Communities that Care (Hawkins & Catalano, 1992). To add to the important recent focus on environmental prevention approaches such as regulating prescribing of opioids, reducing access to opioids, and reducing harms of overdose such as through naloxone distribution and access (Egan et al., 2024) and extend prevention efforts by targeting opioids, the present study aims to understand the potentially unique experiences of opioid use among adolescents and young adults (Fraser & Plescia, 2019; Helme et al., 2020; Volkow et al., 2018) drawing on the stories of those in recovery for opioid use disorder (OUD) (Rigg et al., 2013).

Opioid use and opioid use disorder (OUD) have affected young people from diverse demographic backgrounds across social class, race/ethnicity, gender, and biological sex, with some studies pointing to differences in types of substances that young people are using (e.g., Peteet, 2017; Rigg et al., 2024; Schepis et al., 2018). Extensive evidence about the risk and protective factors for various substances, including the nonmedical use of prescription opioids, points to risk factors such as emotion regulation difficulty, prior substance use, friends and family who use substances, accessibility, and disconnection from institutions (Nargiso et al., 2015; Nawi et al., 2021). Some research suggests that opioid use may be best understood in the context of polysubstance use (Cicero et al., 2020) and prevented with existing evidence-based prevention strategies (Pandika et al., 2022). However, the extent to which young people may have some specific motives for and experiences with using opioids compared to other substances is unclear. This is consequential because different motives may be associated with different experiences and can potentiate different use patterns (Young et al., 2012). Further, insights about unique experiences with opioid use can inform how best to supplement or tailor existing evidenced-based prevention approaches.

Theoretical accounts and reviews suggest potential motives for opioid use such as pain relief (including physical and emotional), experimenting, social influences, recreation, and easy access (Bonar et al., 2020; Remillard et al., 2019; Rigg et al., 2018). Among adolescents, one study revealed that pain relief and getting high were the two most common motives reported for medical misuse of prescription opioids (McCabe et al., 2013). Research examining questions around motives for opioid use typically focuses on recent opioid use (e.g., within the past year) among adults (Miglin et al., 2022; Rigg et al., 2018; Rigg et al., 2024). A missing piece in understanding motives and experiences associated with opioid use is the voice of young people in recovery from OUD. Their retrospective accounts making sense of their motives and experiences with opioid use are highly valuable as they can draw on lived experience of both substance use disorder and recovery journeys, and many have reflected on their own pathways as well as how to support others in prevention spaces. Their accounts can provide key insights to improve opioid use prevention (Rigg et al. 2013). The present study draws on retrospective accounts among young adults in recovery from OUD, from a state in the Southeastern USA, to examine (1) self-reported motives for opioid use and (2) experiences and factors that participants associate with opioid use as compared to other substances.

## Method

### Study Design

We collected both quantitative and qualitative data. Our quantitative data focus on retrospective motives for opioid use; qualitative data explore self-reported factors related to opioid use compared to other substances. The study was conducted under the purview of the Institutional Review Board at [name of institution removed, IRB00054756].

### Sample and Procedure

The sample consists of 30 adolescents and young adults (AYAs) who identified as being in recovery from OUD at the time of recruitment. Participants were recruited using a network-based approach, posting study flyers on recovery-related online groups and platforms, through college recovery programs, formal recovery programs (such as Triangle Residential Options for Substance Abusers), and through a snowball sampling technique by which participants were recruited by their friends. Semi-structured interviews ranging from 15 to 90 min were conducted via videoconferencing between May and October 2021. The interview guide was divided topically into 4 parts, including: current life, substance use history, process of recovery, and ideas for incorporating recovery perspectives into prevention. Surveys were completed afterwards through an online survey tool. The largest proportion of participants identified as female, White, and from suburban areas (see Table 1). Inclusion criteria were: being between the ages of 18 to 29 and identifying that opioids had presented problems in their lives. All individuals identified as being in recovery from substance use disorder, including from opioids, and were required to have a strong social support network and/or sponsor at the time of study participation to buffer potential risks of discomfort or need to process topics that may arise during interviews. Recovery is a broad term with different definitions; we let participants self-identify about being in recovery. Our screening form asked participants “Thinking about your current or past opioid/non-medical prescription opioids use, which best describes you” with the options “currently using, not currently using, in recovery.” All participants self-identified as either “not currently using” opioids (*n* = 2) or being “in recovery” (*n* = 28) and all identified a date they considered the start of the period of recovery for them. Participants followed different recovery pathways (e.g., Twelve Steps, Refuge Recovery); we did not inquire whether participants followed abstinence or medication assisted recovery (MASKED FOR REVIEW). Twenty seven out of 30 individuals reported additional substances as having presented problems and all but one participant identified first using opioids in pill form while one participant reported that their first use of opioids was intravenous.

**Table 1:** Sample demographics

Gender	
Male	10
Female	19
Non-binary	1
Race/ethnicity	
White	23
Black or African American	3
Hispanic, Latino or Spanish origin	1
American Indian or Alaskan Native	3
AAPI	2
More than 1 race	2
Born in USA	27
Not born in USA	3
Age	
18–21	3
22–25	10
26–29	17
Geographic	
Urban	11
Suburban	16
Rural	3
Parental education	
High School/GED	7
Some College	7
College Degree	12
Graduate Degree	4

Note. AAPI, Asian American Pacific Islander; GED, General Education Development

#### Measures

##### Quantitative

Motives for opioid use were assessed using a modified version of the Drinking Motives Questionnaire-Revised (DMQR, Cooper, 1994). In the modified DMQR survey used in the present study, the word “alcohol” was replaced with “opioids”. The original 20-item questionnaire assessed four types of motives to use alcohol: for coping (e.g. “to forget your worries”), conformity (e.g. “to fit in with a group you like”) enhancement (e.g. “because you like the feeling”), and social (e.g. “to be sociable”) reasons.

##### Qualitative

Semi-structured interviews followed our interview guide (MASKED FOR REVIEW) asking about participants’ current life, reflections regarding participants’ substance use (specifically their experiences with opioids), experiences with recovery, and suggestions for prevention.

#### Analysis

##### Quantitative

We conducted descriptive statistical analysis of the surveys to summarize motives for opioid use. We created composite scores of the DMQR subscales, computed scale reliabilities using Cronbach’s alphas, and computed means and variance (Table 2).

**Table 2:** Retrospective self-reported motives for opioid use

Variable name	# Items	Reliability	*N*	Mean	SD	Min	Max
*Motivations to use*							
DMQR enhance	5	0.832	30	4.393	0.826	1	5
DMQR coping	5	0.863	30	4.367	0.830	1	5
DMQR social	5	0.815	30	3.587	1.066	1	5
DMQR conform	5	0.720	30	1.930	0.850	1	5

Note. DMQR = Drinking Motives Questionnaire-Revised. The measure was modified in the present study to ask about motives for opioid use

##### Qualitative

**Fig. 1:**
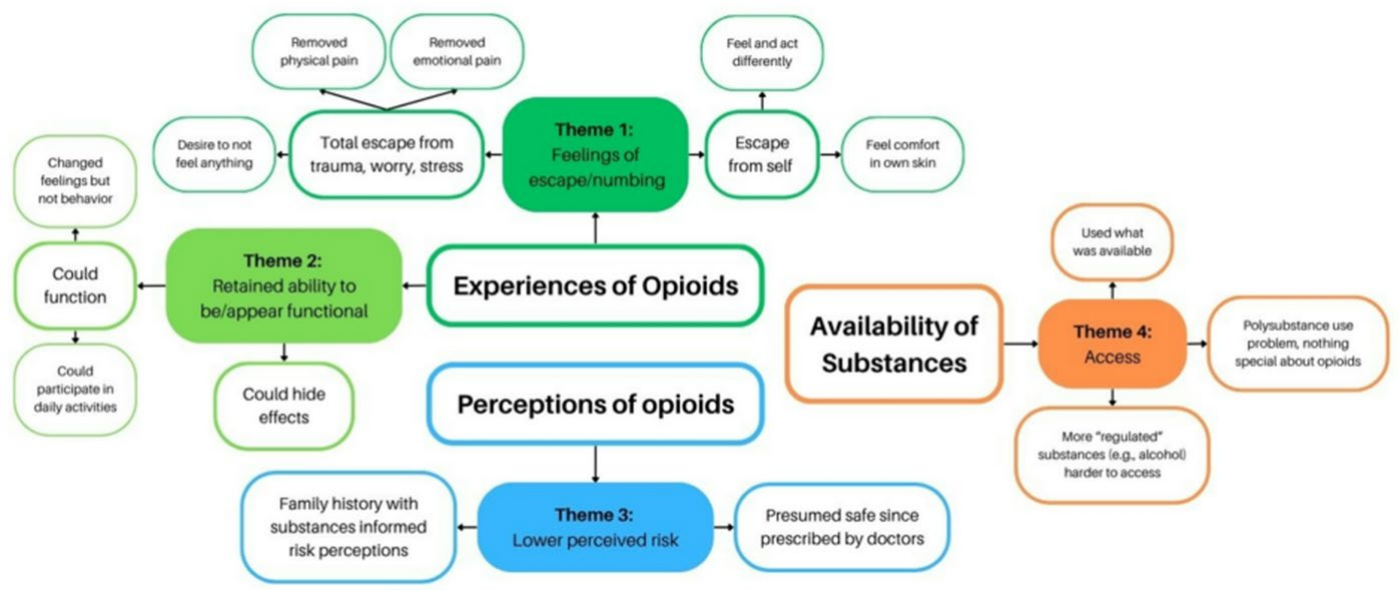
Visual representation of findings. Themes (shaded squares) constructed from examples (unshaded)

Interviews were analyzed through a series of steps following a thematic analysis approach and utilizing inductive coding (Braun & Clarke, 2006; Terry et al., 2017). We approached interview data analysis using the six phases of TA outlined in Terry et al. (2017): familiarizing ourselves with the data, generating initial codes, searching for themes, reviewing themes, defining and naming themes, and generating a report (Braun & Clarke, 2006; Braun et al., 2022; Terry et al., 2017). When generating codes, we iteratively and recursively developed a list of codes collating the data to specific conceptual ideas as a way to organize data. Consistent with the TA analysis model we followed, and with the approach more recently specified as Reflexive Thematic Analysis (RTA, Braun & Clarke, 2023), we did not conduct tests of inter-rater reliability or use codes as a way to quantify data. Rather, multiple team members applied codes to the text in a process meant to structure discussions and facilitate the next analytic step in which the research team constructed interpretative themes through group discussions (See Fig. 1 for a visual representation of themes as constructed from examples). Throughout this process– from the interview guide design, through data collection, to data analysis– our team individually wrote memos reflecting on our positionality and held reflective workshops to discuss our own subjectivity and relation to the data and broader research topic. This was crucial to our design and interpretation of data as many team members do not identify as being in recovery from substance use disorder. Please refer to (MASKED FOR REVIEW) for more description of the qualitative analysis and positionality of the research team. Interviews were conducted once, and findings were shared with participants (MASKED FOR REVIEW) for additional detail about data collection procedures and process for sharing findings back with study participants).

## Results

### Quantitative

The most endorsed motives for opioid use were using opioids for *enhancement* (i.e., trying to have more fun or excitement; *M =* 4.39, *sd* = 0.83) and for *coping* (i.e., to forget worries or cheer up when in a bad mood; *M =* 4.37, *sd =* 0.83) reasons, followed by *social* motives (*M* = 3.59, *sd* = 1.07), with very low endorsement of *conformity* motives (*M* = 1,93, *sd* = 0.85). See Table 2.

### Qualitative

We identified four main themes regarding potentially unique aspects of opioid use compared to other substances. Overwhelmingly, the *experience* of opioid use seemed to differ from that of other substances, with effects described in terms of feelings of escape and numbing (theme 1); and the ability (early in trajectories of opioid use) to remain/appear functional (theme 2), specifically while under the influence of opioids. In addition, a unique experience related to opioids was perceiving low risk of harm compared to perceptions of risk/harm associated with using other substances (theme 3). Finally, we identified that for some participants, there was nothing particularly unique about opioids and instead environmental *access* was the main factor motivating use (theme 4, Table 3). We conducted qualitative coding in a parallel process to quantitative analyses to capture themes that we interpreted from the data; however, below we note where the themes we coded directly supplement the quantitative motives.

**Table 3:** Themes and illustrative quotes describing experiences of opioid use

Theme	Illustrative Quote
1. Feelings of escape and numbing	*“I know I had a lot of things going on as a kid*,* like mental health-wise. And over time*,* doctors just told me*,* “Well*,* you’re self-medicating*,*” this*,* that*,* and the third. But in a way*,* I was trying to escape reality”* (118)
2. Retained their ability to be/appear functional	*“…but with the booze*,* you got to a point where you couldn’t even speak*,* and couldn’t talk*,* walk…with weed*,* it was more paranoid*,* and not being able to talk. But when I hit pills*,* it was one of those things where I was able to have conversation”* (116)
3. Lower perceived risk of opioids	*“And because it was introduced to me in such a way that it seemed okay for me to use it*,* it was suddenly okay for me to steal it from other people*,* and to use it in excess. In my teenaged mind*,* it made sense*,* y’know? “This is prescribed from doctors*,* so this can’t be bad for you”* (106)
4. Access	*“I got to say that it was way harder to get alcohol than it was to get drugs because you had to go to a beer distributor or a wine and spirits….”* (103)

#### Theme 1: Experiences of Opioids - Feelings of Escape and Numbing

In terms of using opioids as an escape, many participants described their experiences as allowing them to not feel pain, especially emotional pain or negative feelings, or to feel numb. “It numbed the physical pain. It numbed the emotional pain. It numbs all of it. At the end of the day, that’s really all I wanted was to just not feel the way that I was feeling” (102). The particular experiences that participants were trying to numb or escape from varied; many reported that they wanted to escape from dealing with trauma, worry, stress, and/or anxiety. Comparing opioids to other substances, one participant described:

“I feel like Xanax makes you forget things but opioids make you never even have to remember the things. Like you can forget things whenever you do Xanax. Like you’ll forget that you did something. But whenever you go [on] any kind of opiate like you’re just so numb that you don’t even have to remember what you’re trying to forget” (120).

This distinction seems to suggest more than just a higher degree of numbing when taking opiates compared to other substances, but a categorically different experience of total escape from problems or issues that one may want to escape from. Another participant similarly described the escape that using opioids provided in terms of numbing, “Well with the opioids the reason why I was so addicted to them is because they numb you so much.” The participant continued, “It’s truly just like you don’t have to feel anything when you take an opiate. It takes away those worries and pain and emotions. It just takes it all away so you don’t feel anything” (107). For these participants, it seems to be the *complete* ability to be numb to any feelings, especially negative ones, as a result of opioid use that may have differed from their experiences with other substances which may have provided a partial escape. These examples align with the quantitative data pointing to a main motive of opioid use being to cope with negative feelings and difficult experiences as many forms of escape were meant to cope with emotional and traumatic experiences.

Other participants discussed escape in terms of escaping from being themselves as opioids allowed them to feel and act differently. When asked to compare opioids to other substances, one participant explained that “there’s no other comparison. I’ve done a lot of drugs in my lifetime and opioids, and anything associated with them, from fentanyl down to Vicodin, there’s nothing that compares to it” and elaborated:

“Initially, energy. I was social. I was able to talk to people. I could have a good time. Since I wasn’t very social, I didn’t– enjoyment for me in life was video games. That was my enjoyment. Not interpersonal contact. Through shyness, embarrassment of being a young person, how I looked, body, you know, like what normal people go through. I found something, at that time in my life, that dissolved those feelings. It made it to where I didn’t care…I was able to have more fun. I was able to enjoy life” (119).

For this participant, opioids allowed them to escape from being himself and “dissolved” difficult feelings and self-perceptions and ultimately allowed him to be social and enjoy himself. Similarly, another participant described how opioids allowed him to escape from being himself by taking the edge off social interactions. He explained that when using opioids, “I felt comfortable being around people. I wasn’t scared. I wasn’t shy… It’s like Batman puts on his cape before he goes out; I do pills before I go out, so I can mingle, basically” (116). Notably, three out of the 30 participants described that opioids allowed them to feel “comfortable in their own skin,” the specific use of this exact phrase suggests this as a major function of opioids that motivated continued use among participants. For some participants, the experience of opioids was described slightly differently, as highly pleasurable in terms like “euphoria” (108, 115), “calmness” (103), and “peace” (101). Overall, these experiences with opioids, which seem to be described in terms of numbing and escape, align with quantitative findings supporting enhancement motives (such as creating the conditions for more fun and excitement experiences via escaping from negative feelings and perceptions of oneself) and coping (especially with negative feelings) motives for opioid use.

#### Theme 2: Experiences of Opioids - Retained Ability To Be/Appear Functional

Many participants described how opioids were different from other substances because they *retained the ability to be/appear functional* (theme 2) while under the influence of opioids. This was explained both in terms of being able to use opioids and still participate in daily activities (e.g., at home, school, or work) and in terms of masking the effects of substances (e.g., not appearing to be high). Several participants discussed how they could not function when using other substances, especially alcohol and Xanax, but opioids let them feel high while also still able to participate in activities in their daily lives. For example, one participant explained “Coming to school drunk or stoned was too obvious…but opiates and coming to school, it felt different. I felt like I could still function, but I just felt really good” (104) Some participants further explained how others (e.g., their parents or teachers) could not tell if they were using opioids, whereas it was more obvious when they were using other substances. One participant transitioned to opioid use when they had to stop using marijuana due to legal trouble; he explained that it was easier to work around drug tests with opioids, “…I figured opiates only stay in your system six days. I know when I have to go to the office and he doesn’t drug test me at home. So, if I do opiates and stop a week before, I’d be okay” (102). Thus, appearing functional and passing drug tests were specifically identified by participants as perceived benefits of opioids compared to other substances. We observed this theme from our qualitative data and contend that this is a potentially unique motive compared to other substances; the items in our quantitative coping measure did not capture this motive for opioid use.

#### Theme 3: Lower Perceived Risk of Opioids

Whereas the examples above illustrate how the *experience* of opioids differed from those of other substances, one interesting point raised by participants was about the *perceived risk* of opioids related to other substances (theme 3). Two participants expressed that opioids (in pill form) seemed less risky because they are prescribed by doctors. For example, one participant described, “…growing up, it was always ‘don’t drink and don’t do drugs,’ but drugs is so broad that sometimes you don’t really think of a painkiller to be a drug” (109). Similarly, another participant was aware of the risks of addiction since her father had issues with alcohol. Due to less emphasis placed on opioids compared to other substances in her home, combined with the fact that they were prescribed by doctors, she did not perceive the risk to be as high:

“My mom was really scared. She was really, really scared that I would end up like my dad, and have an issue with alcohol. So she used to like tell me it was the devil’s poison, and really put the fear of God into me about using like marijuana and alcohol specifically. So I didn’t see any issues with using prescription medications that were prescribed to me, ‘cause those were from doctors. What’s wrong with those, y’know? They’re supposed to help you” (106).

For these participants, discussions at home centered on risks associated with some substances more than others (e.g. opioids); this may have combined with knowledge that “doctors prescribe pills” to reduce overall perceived risk of opioids in pill form. Overall, the three themes we identified above capture several ways in which participants articulated how the *experiences* and *perceived risks* for opioid use, especially in pill form, differed from other substances.

#### Theme 4: Access

For some participants, there was nothing different or special about opioids; rather, they described their opioid use as a matter of environmental access (theme 4) and what was available to them in a given place at a given time. One participant said, “but for me, it was so much of a polysubstance abuse problem that I really did not differentiate and discriminate” (105). Several participants noted the role of access: for some, opioids were easier to access compared to other substances; this may have been especially true for opioids not in a pill form. One participant noted, “from the region I am in Eastern North Carolina, it was easier to get opioids than it was alcohol” (119). Another participant noted, “where I grew up at wasn’t the best parts, and so I could walk outside, and it was a rough neighborhood.” He explained, “And me being 12 years old, I could walk down the street and buy a bag of cocaine and a bag of marijuana if I wanted it at that time. Definitely where I grew up at had a big impact” (118). For many others, opioids were difficult to access and the source who gave them opioids was an important factor in why they tried/liked the experience. Several female participants specifically noted gaining access to opioids (in both pill and non-pill forms) through romantic partners. Thus, general issues related to substance access as well as specific access issues related to sources of opioids were relevant factors for many participants’ opioid use.

## Discussion

Participants reported that their experiences and perceptions of opioids differed somewhat in specific ways compared to their experiences with, and perceptions of, other substances. The two top motives for opioid use reported on the surveys were *coping* and *enhancement.* This aligns with a large literature pointing to coping with stress as a main motivator of substance use among adolescents (Boke et al., 2019; Sinha, 2008). However, this differs somewhat from the motives reported for other substance use, such as alcohol, where the most common motives tend to be social (Bradizza et al., 1999; Kuntsche et al., 2005), although enhancement and coping motives for alcohol may be related to more problematic drinking (Kuntsche et al., 2005; Kougiali et al. 2021). In our qualitative analyses, participants noted that opioids provided a feeling of escape from dealing with trauma, such as emotional neglect and other events, as well as an escape from dealing with negative emotions. This is broadly aligned with self-medication and related hypotheses regarding motives for substance use although the current retrospective study design in unable to test for the direction of effects (Broman et al., 2019). This finding aligns with qualitative studies on opioid use initiation, which point to opioid use to cope with complex psychological and emotional issues as well as adverse childhood experiences, trauma, social influences, and life stressors (MASKED FOR REVIEW, Bonar et al., 2020; Cicero & Ellis, 2017; Forston et al., 2021; Regmi et al., 2024; Remillard et al., 2019; Rigg et al., 2018; Rigg et al., 2024). Coping with trauma is also highly endorsed among women dependent on alcohol, with a recent review finding that sexual and physical trauma are common experiences motivating women’s initiation and maintenance of alcohol use (Kougiali et al. 2021). Trauma was also reported by participants in the present study; however, emotional neglect was the most endorsed form of trauma. Findings also support the 2021 advisory notice from the Office of the Surgeon General on the need for mental health care and coping skills, which emphasized the importance of mental health care screening and access as a prevention strategy (Office of the Surgeon General, 2021; Ridenour et al., 2021), especially given the comorbidity of substance use disorders with mental health issues and disorders (Köck et al., 2022; Yule et al., 2023). Our findings extend this work by examining motives among individuals who are in recovery from OUD whereas most prior work has focused on opioid use initiation. Ideally, structural solutions would result in young people experiencing less trauma and stress and experiencing more support and connection to deal with normative negative emotional experiences (MASKED FOR REVIEW).

In addition to coping with negative emotions, some participants perceived that opioid use gave a temporary feeling of confidence in their social skills (aligned with enhancement motives) and allowed the ability to be functional in everyday life including interactions with family, at school, and at work. One review of studies with adult samples of women with alcohol use disorder, participants reported a motive for alcohol use was the ability to maintain functionality (Kougiali et al. 2021), although it is interesting that a couple of participants in our sample specifically noted that appearing functional was not possible when using alcohol. This might be context-specific with the current sample of young adults focused on being functional while performing tasks in school or work while alcohol was noted to increase functionality in social situations (Kougiali et al. 2021). This finding may point to a future motive for opioid use that is not often captured though survey measures of motives for using some substances over others.

Some participants discussed how the experience of opioids allowed them to escape feeling like themselves and to shed their feelings of discomfort temporarily (even though longer-term consequences were detrimental to their sense of self). Given that some discomfort with self is common during adolescence as young people undergo identity exploration and transformation (Branje, 2022), it is crucial that future research examines the individual and contextual factors that render general discomfort with oneself a risk factor for some to use substances to cope. The finding that negative emotions and difficult experiences are motives for substance use align with past research (Bonar et al., 2020; Cicero & Ellis, 2017; Remillard et al., 2019; Rigg et al., 2018). Notably, in the present study, these came up in the context of comparing the experience of opioid use and the relief it provided participants to experiences with other substances. Thus, our findings support the call for screening for opioid use risk in the contexts where young people are receiving mental health services (Yule et al., 2023).

In addition to the *experience* of opioids being different from other substances for many participants, we found that *perceptions of* opioids were different. Specifically, opioids taken in pill form were perceived as less risky compared to other substances such as alcohol because they are prescribed by doctors, and in some cases, their risks may be less emphasized by parents. This is an important finding. Although this may be less true today than in earlier phases of the opioid epidemic, the finding aligns with recent evidence that opioids in pill forms are perceived as less risky than other forms (Evans et al., 2020). The non-medical use of prescription opioids is a known pathway to OUD (Baldwin et al., 2021; Volkow et al., 2018) and many prevention policies and efforts are now in place to combat over-prescribing of pain medications (e.g., Lock Your Meds). However, supplemental prevention approaches may be needed that target opioids. This is especially relevant in the modern context where pills are increasingly likely to be laced with fentanyl and other drugs or adulterants (DEA, ND; Singh et al., 2020).

Opioids were used alongside other substances by all but three participants (MASKED FOR REVIEW) and for some participants, issues related to access were primary in their descriptions of motives for opioid use. Indeed, one participant specifically noted that opioids were part of a larger polysubstance use problem, and more than one participant noted that easy access to opioids was the main factor that led to their use, in contrast to unique perceptions about or experiences with opioids. In most cases, the first substance used was reported to be alcohol or marijuana while a few participants described use that began with prescription opioids. This aligns with findings regarding use patterns that urge consideration of polysubstance use patterns rather than focusing on one substance at a time (e.g., Cicero et al., 2020; Crane et al., 2021) and emphasize the shared risk factors for opioids and other substances (Pandika et al., 2022). While retaining a focus on evidenced-based strategies for general substance use, which may be the most effective prevention approach (Pandika et al., 2022; Stockings et al., 2016), our findings suggest the importance of incorporating specific content aimed at educating about the harms associated with opioid use given potentially unique experiences associated with opioids and lower perceived harms.

### Limitations

Characteristics of the study design and sample should be considered when interpreting results. The sample was mostly female, suburban, and white, which may not reflect the broader experiences of YAs in recovery in NC. This is a sample of YAs who identified as being in recovery from OUD and most of them identified additional substances that were problematic for them. Although we asked specifically about opioid use, without a comparison group we cannot directly compare experiences and perceptions of opioids with those associated with using different substances. Also, retrospective self-reports are subject to potential biases associated with individuals’ memories and life events in the intervening years between opioid use and study participation. Specifically, this sample of YAs in recovery have extensive experience reflecting on their substance use and discussing their use and their lives through recovery programs and in many cases through mental health counseling and therapy, thus their language and the way they made meaning of their experiences may be influenced by the frame of recovery. Our inclusion criteria requiring that participants have active support (through recovery services or social support) to be in our study may especially make this sample different from those in recovery but without current social support; they may have more practice discussing their experiences and may also have systematically different experiences in terms of their motivations compared to those without social support networks. Still, the voices of young adults in recovery from OUD provide valuable insights about how people perceive, and experience opioids and these insights should inform prevention. Understanding opioid use motives among individuals in recovery for OUD might help identify those who are at greater risk of moving beyond initiating opioid use to developing OUD. This information could supplement current risk assessment tools by including questions that capture motives for use, experiences of opioid use, and perceived risks associated with opioid use. Finally, it is important to note that the retrospective accounts in the present study pertain to earlier in the opioid epidemic; motives and experiences with opioid use may have changed over time as the epidemic has continued to shift, for example, perceptions of the risks associated with prescription opioids is likely higher now. In addition, we adapted an alcohol motive questionnaire that matched the items we intended to capture; specific opioid motive questionnaires will be helpful for research in this area.

### Implications for Prevention

Our findings have implications for the continuum of preventing opioid use (Cance et al., 2023). In terms of universal prevention, our findings support “upstream” approaches that focus on mental health screening and support as well as building coping skills, specifically coping with negative emotions. Such prevention approaches might be especially useful during early adolescence, a developmental time of growth and identity formation. Evidence-based prevention programs (Pandika et al., 2022), such as Life Skills Training and Coping Power may be promising (Botvin & Griffin, 2004; Lochman & Wells, 2003). In addition, supplemental or tailored approaches or messaging are needed to focus on misperceptions among some young people that prescription medications confer low risk compared to other substances. For example, mass media campaigns, such as The Truth About Opioids, show promising effects on increasing knowledge about the risks of opioids among young YAs (Rath et al., 2022). There is much greater awareness now about the risks of opioids compared to several years ago when present study participants were adolescents; however, it may be useful to tailor specific messaging to young people about the risks of substances such as opioids that are sometimes prescribed by trusted experts and are safe in some contexts (Cance et al., 2023). Continued environmental prevention strategies are needed to limit access to opioids.

For selected and indicated prevention approaches, it will be important to address mental health risk factors as motivations for opioid use seem to be related to coping. The finding that people may be/appear more functional when using opioids compared to other substances suggests that early detection of opioid misuse may be more difficult compared to other substances, necessitating that adults establish and maintain open communication with young people and that risk assessments specifically ask about different substance use behaviors and experiences. Among those who have tried opioids and perceive the ability to remain functional for daily activities, it may be helpful to emphasize the long-term functional harms associated with opioids. Overall, existing evidence-based environmental and individual prevention strategies are promising; tailoring programs to curb access to opioids, increase mental health access and support related to coping strategies, and tailoring messages specific to the harms associated with opioids may benefit prevention of opioid use disorder.
